# Direct Fitness Correlates and Thermal Consequences of Facultative Aggregation in a Desert Lizard

**DOI:** 10.1371/journal.pone.0040866

**Published:** 2012-07-23

**Authors:** Alison R. Davis Rabosky, Ammon Corl, Heather E. M. Liwanag, Yann Surget-Groba, Barry Sinervo

**Affiliations:** 1 Department of Ecology and Evolutionary Biology, University of California Santa Cruz, Santa Cruz, California, United States of America; 2 Department of Integrative Biology and Museum of Vertebrate Zoology, University of California, Berkeley, California, United States of America; 3 Department of Ecology and Evolutionary Biology, University of Michigan, Ann Arbor, Michigan, United States of America; 4 Department of Evolutionary Biology, Evolutionary Biology Centre, Uppsala University, Uppsala, Sweden; 5 Department of Biology, Adelphi University, Garden City, New York, United States of America; 6 Ecological Evolution Group, Xishuangbanna Tropical Botanical Garden, Menglun, Mengla, Yunnan, P. R. China; University of Manitoba, Canada

## Abstract

Social aggregation is a common behavioral phenomenon thought to evolve through adaptive benefits to group living. Comparing fitness differences between aggregated and solitary individuals in nature – necessary to infer an evolutionary benefit to living in groups – has proven difficult because communally-living species tend to be obligately social and behaviorally complex. However, these differences and the mechanisms driving them are critical to understanding how solitary individuals transition to group living, as well as how and why nascent social systems change over time. Here we demonstrate that facultative aggregation in a reptile (the Desert Night Lizard, *Xantusia vigilis*) confers direct reproductive success and survival advantages and that thermal benefits of winter huddling disproportionately benefit small juveniles, which can favor delayed dispersal of offspring and the formation of kin groups. Using climate projection models, however, we estimate that future aggregation in night lizards could decline more than 50% due to warmer temperatures. Our results support the theory that transitions to group living arise from direct benefits to social individuals and offer a clear mechanism for the origin of kin groups through juvenile philopatry. The temperature dependence of aggregation in this and other taxa suggests that environmental variation may be a powerful but underappreciated force in the rapid transition between social and solitary behavior.

## Introduction

Few topics within evolutionary biology have inspired more excitement and controversy than the origin and evolution of social behavior [1], [2], [3], [4]. The tendency for individuals to form conspicuous groups is surprisingly widespread and easy to observe across both vertebrates and invertebrates, with repeated independent origins throughout many taxa [5], [6], [7], [8], [9], [10]. Sociality can take many forms, from the simple clustering of individuals in space to complex forms of cooperative breeding and reciprocal altruism [11]. However, one of the most poorly understood aspects of the evolution of any social system is the origin of group interaction in a population of solitary individuals [5]. Regardless of the eventual complexity of a social system, the evolutionary transition from solitary living to any form of sociality must begin with the simple act of initiating and maintaining contact with conspecifics, which is best described as aggregation.

Social aggregation is thought to evolve though adaptive benefits to group living, but hypothesized benefits of communal behavior can be difficult to quantify, compare, and interpret in natural systems. Most empirical research on social evolution has focused on taxa with obligate sociality (eusocial insects, most birds and mammals) and highly complex social interactions, precluding comparison to solitary individuals and limiting insights to the maintenance of sociality rather than its origins [12]. Thus, the mechanisms and directionality of fitness advantages to group living remain contentious but critical to understanding why solitary individuals transition to communal living and how group formation can change over time [13], [14], [15], [16].

Comparative studies across social insects [17], spiders [18], birds [7], mammals [6], fish [19], and lizards [8], [9] have revealed that many groups form specifically through delayed dispersal of offspring, creating highly related kin groups [20]. The broad taxonomic distribution of this pattern suggests that extending the interaction of parents and offspring is a simple, common process by which solitary animals become social [8], [20]. However, the ecological mechanisms promoting long-term juvenile philopatry are rarely tested empirically (but see evidence in cooperatively-breeding birds [21] and fish [22]). Hypothesized mechanisms tend to be species-specific and invoke complicated group cooperation scenarios, but several suggest dependence on resource availability or environmental conditions (biotic or abiotic) that are inherently unstable [20], [21], [23]. Directly testing these mechanisms offers a powerful way to understand how kin groups form by explaining why juveniles may drive the transition from solitary to group living and by predicting when and how this behavior may change as environmental conditions vary. This integration of ultimate fitness consequences of social behavior with the proximate mechanisms promoting aggregation among kin [13] is key to understanding how and why social systems may form and change over time.

Here we show direct empirical evidence that social group participation in a reptile (the Desert Night Lizard, *Xantusia vigilis*) provides both survival and reproductive success advantages, test a physiological mechanism driving these fitness benefits, and predict changes to social behavior with changing environmental conditions. *Xantusia vigilis* is a very small (1.5 g), long-lived (at least 8–10 years), viviparous lizard that lives at high densities under fallen logs (*Yucca sp.*) in the Mojave Desert of California, USA [24], [25]. Night lizards are highly secretive and rarely seen away from cover objects. Despite their common name, night lizards are diurnally active, especially in the winter [26].

Every winter between November and February, night lizards aggregate into groups of 2–20 individuals underneath fallen logs (Figure S1). Molecular analyses and field studies have previously shown that these aggregations are often highly related family groups produced through delayed juvenile dispersal, although groups without juveniles can contain kin or unrelated individuals [8]. Cross-fostering manipulations show that juveniles remain philopatric and aggregate specifically when with kin as opposed to unrelated individuals, so these family groups do not form simply as a by-product of globally infrequent dispersal [27]. Furthermore, groups of related individuals are stable across years despite aggregation being a winter-restricted phenomenon, as individuals will re-select former aggregation partners from a high density pool after eight months of solitary living during the spring, summer, and early fall [8]. These groups form outside of the mating (May–June) or birthing (August–September) seasons and independently of resource distribution and habitat quality, as there are no environmental differences between sites containing aggregations as opposed to solitary lizards (Figure S2–S3; Text S1; [8]). However, only about 2/3 of the population participates in aggregations each winter [8], and this facultative behavior allows the rare comparison between aggregated and solitary individuals and a test of mechanisms promoting social group formation.

We used this natural variation in social behavior for three specific tests. First, we compared fitness measurements between naturally aggregated and solitary individuals in the field to test for adaptive benefits encouraging the formation of social groups. Second, we examined the temperature-dependence of aggregation behavior and compared rates of heat loss between solitary lizards and aggregations to test a thermal mechanism driving juveniles to join groups. Third, we used climate models to predict how this temperature-dependent aggregation will respond to changing environmental conditions, informing how social behavior can change over time.

## Results

**Figure 1:**
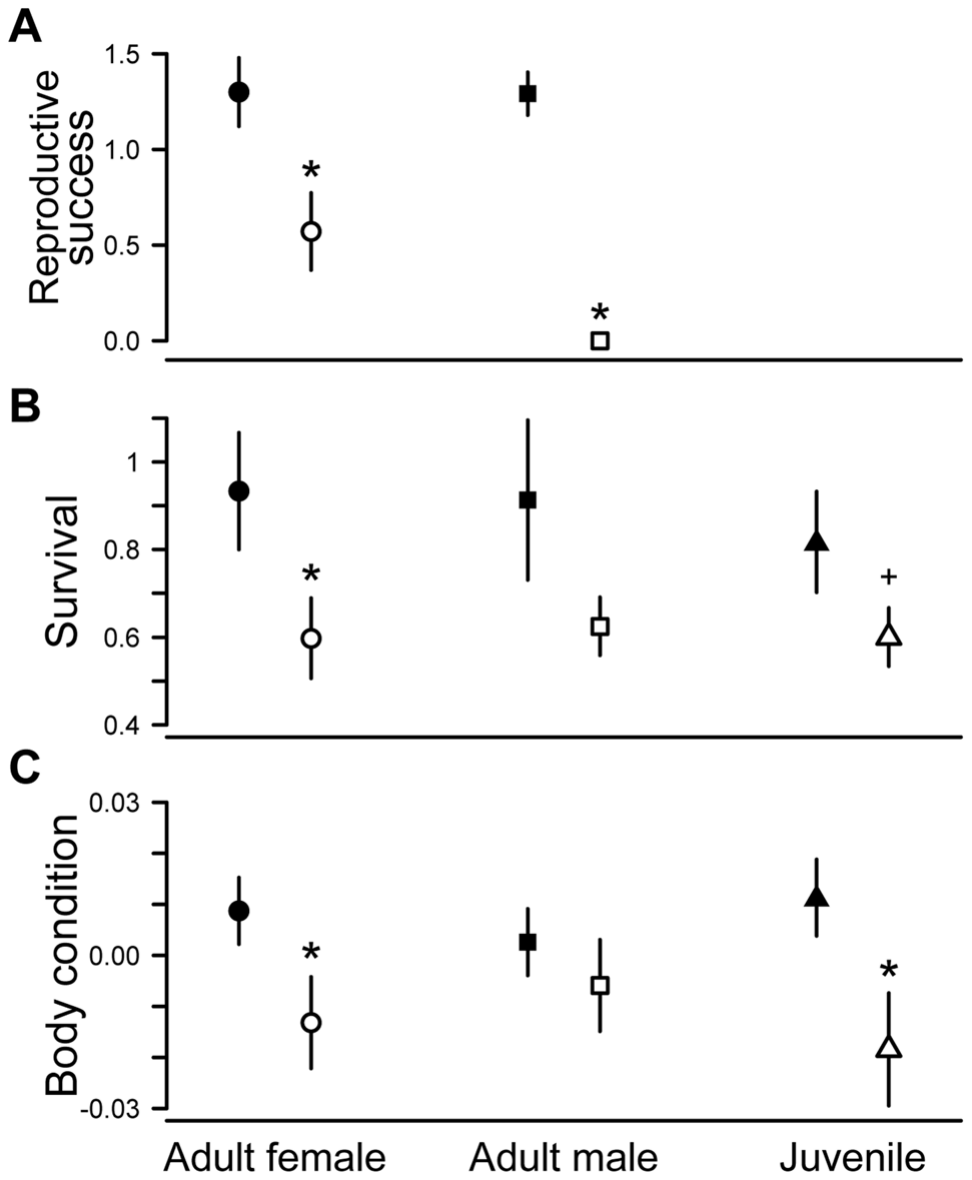
Fitness comparisons of aggregated and solitary night lizards. Higher fitness of aggregated (filled symbols) versus solitary (open symbols) lizards shows clear adaptive benefits to winter aggregation (circles  =  females, squares  =  males, triangles  =  juveniles). (**A**) Reproductive success (# offspring) of lizards that were aggregated or solitary in the winter directly preceding summer reproduction (*N_F_* = 34, *N_M_* = 24; see text). (**B**) Multi-state model estimates of survival associated with aggregated and solitary social states (*N_F_* = 723, *N_M_* = 443, *N_J_* = 1166). (**C**) Body condition (residual mass on body size) of all winter-collected lizards by aggregation state (*N_F_* = 640, *N_M_* = 389, *N_J_* = 507). Asterisks and cross denote *P*<0.05 and *P*<0.1 significance levels, respectively, and error bars are ±1SEM.

To test for reproductive success and survival consequences of aggregation, we combined a mark-recapture field study of 2,332 lizards in 441 social groups with a DNA microsatellite analysis of parentage. For both males and females, we found that lizards caught in winter aggregations had higher reproductive success in the following summer than solitary individuals (females: *t* = 2.70, df = 32, *P* = 0.011; males: x = 24, n = 24, *P*<0.001; Figure 1A). In males, this reproductive skew was extreme, and no winter-solitary male was ever found to sire offspring in a consecutive summer, while several winter-aggregated males sired offspring by multiple females within a single reproductive season. We also found higher survival of aggregated individuals in adult females (χ^2^ = 4.31, df = 1, *P* = 0.038) and a trend in the same direction for juveniles (χ^2^ = 2.82, df = 1, *P* = 0.093) and adult males (χ^2^ = 2.20, df = 1, *P* = 0.138; Figure 1B), which are significant when combined with a weighted Z-test ([28]; *P* = 0.0116; Table S1). Additionally, we found that aggregated lizards had better body condition (plumper per unit body length) than solitary lizards, although this effect was not significant in adult males (females: *F*_1,638_ = 4.07, *P* = 0.04; males: *F*_1,387_ = 0.398, *P* = 0.53; juveniles: *F*_1,505_ = 7.15, *P* = 0.008; Figure 1C).

**Figure 2:**
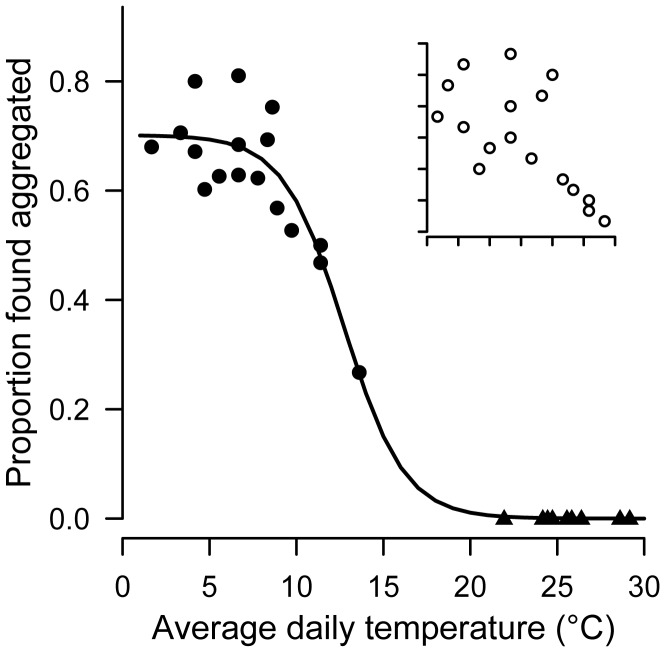
Social aggregation as a function of environmental temperature. The proportion of total lizards found aggregated each field collection day shows strong temperature-dependence (*N* = 17 winter [circles] and *N* = 9 summer [triangles] days; sigmoidal function with both winter and summer points was only used to generate predictions of aggregation levels at unobserved temperatures, see Methods and Figure 4). Inset shows Spearman correlation among ranked winter days only (*ρ* = −0.63, *P* = 0.007).

As aggregation was only observed during winter, we next examined the role of environmental temperature in the prevalence of this behavior (see Text S1 for tests and discussion of other non-significant environmental variables). We found that group formation was strongly temperature-dependent, with high levels of aggregation occurring only on cold winter days (Figure 2 inset, with winter only observations; *ρ* = −0.63, N = 17, *P* = 0.007; sigmoidal function with both winter and summer points was only used to generate predictions of aggregation levels at unobserved temperatures, see Methods). Daily observed aggregation levels did not depend on the total number of lizards caught (*P* = 0.21) and were not an artifact of small-scale fluctuations in daytime temperature (Figure S4).

**Figure 3:**
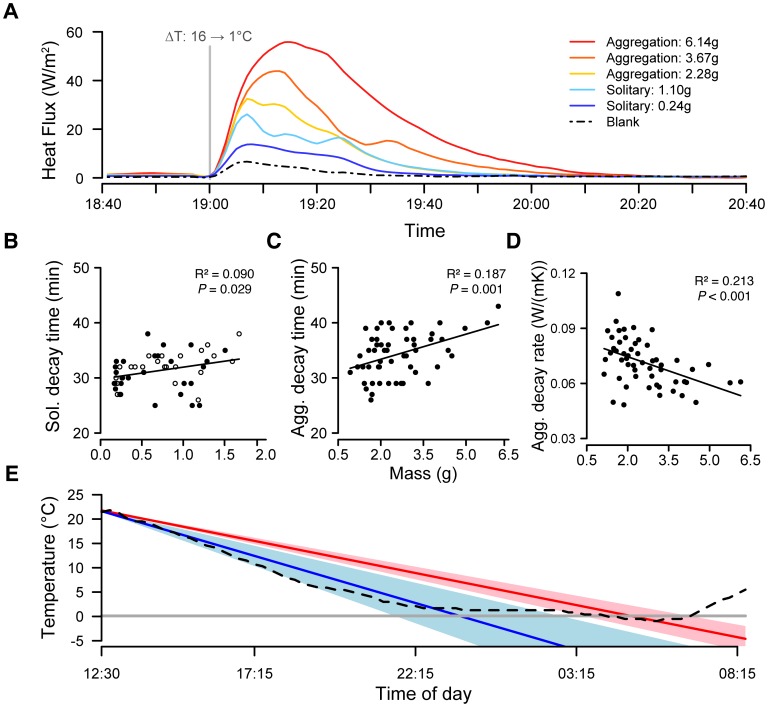
Rates of heat loss in solitary and aggregated night lizards. (**A**) Representative heat loss curves measured for two solitary lizards and three aggregations of different mass show that thermal stability increases with mass because lizards in large aggregations take longer than solitary lizards to return to equilibrium with environmental temperature. Properties of each curve are represented by single points in panels B–D. (**B**) Thermal decay time (two half-lives) for solitary lizards increases with mass (F_1,51_ = 5.07, *P* = 0.029). There was no difference between naturally solitary (closed circles) and experimentally isolated (open circles) lizards (F_1,50_ = 0.59, *P* = 0.45). (**C**) Thermal decay time (two half-lives) for lizard aggregations increases with mass (F_1,53_ = 12.16, *P*<0.001). (**D**) Rates of thermal decay (heat loss) decrease as aggregation mass increases (F_1,52_ = 14.08, *P*<0.001). (**E**) Rates of heat loss under natural conditions, as predicted from measured laboratory rates in (D), show that solitary neonates (blue) reach equilibrium with microclimate temperature (dashed black line; intersection) much earlier than aggregations greater than 4g (red) and spend more time at cold temperatures. Shading denotes ±1 SD, and gray line at 0°C shows environmental freezing point.

Due to the basic principles of convective heat transfer and the fact that huddling physically alters surface area to volume ratios, there are unavoidable thermal consequences to winter aggregation in night lizards. We quantified these consequences by comparing heat flux in solitary individuals, natural aggregations, and experimentally isolated lizards. We found that, as expected, aggregations cool more slowly than solitary lizards (Figure 3A; decay time for solitary lizards: F_1,51_ = 5.07, *P* = 0.029, Figure 3B; decay time for aggregations: F_1,53_ = 12.16, *P*<0.001, Figure 3C; decay rate for aggregations: F_1,52_ = 14.08, *P*<0.001, Figure 3D). This relationship scales positively with mass, such that small juveniles track environmental temperature very closely and derive the greatest increase in thermal stability from social behavior (Figure 3A; note comparison to blank [empty arena] showing environmental temperature). Small-massed juveniles joining aggregations take 30–60% longer to reach equilibrium with environmental temperature than solitary juveniles (Figure 3A–C). When comparing solitary neonates to aggregations under natural field conditions, this heat loss difference translates into an average time lag to temperature equilibrium of about 6.5 hours during a typical night of cooling, and aggregations only experience the coldest temperatures for a limited amount of time (Figure 3E; solitary mean rate = −0.484, aggregation mean rate = −0.329). As winter temperatures in this habitat regularly drop below freezing (Table S2), even underneath fallen logs (Figure S3), the thermal buffer of aggregation is critical to avoiding mortality from freezing and the metabolically expensive tissue repair associated with even mild freeze events (Supporting Information; [29]).

**Figure 4:**
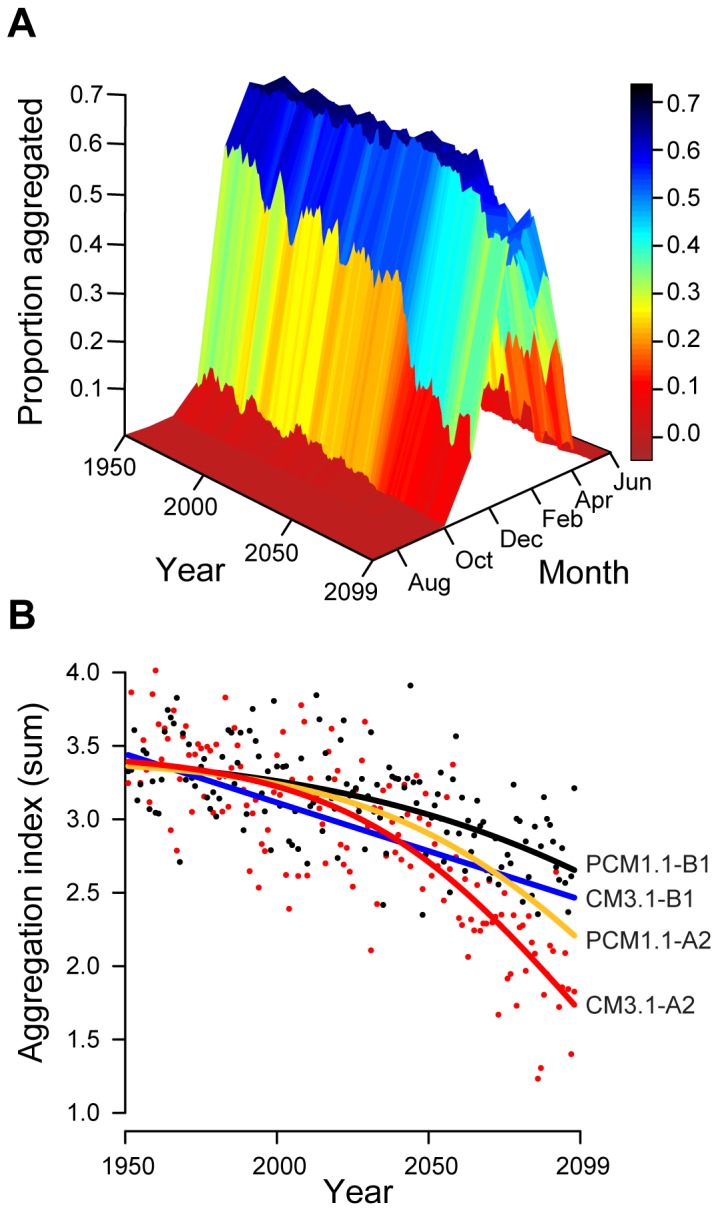
Predicted changes in night lizard aggregation under projected climate scenarios. (**A**) Proportion of the population predicted to aggregate under a climate projection model (CNRM CM3.1-A2). Over time, this proportion decreases both in peak magnitude and in number of months per year with high levels of aggregation. (**B**) Sum of monthly aggregation proportions by year (aggregation index) under two climate models (CNRM CM3.1 and NCAR PCM1.1) and two emissions projections (A2 and B1). Predicted aggregation declines by 20% under mild climate projections and more than 50% under more severe projections (four model mean = 33%). Points generating the curves are shown only for the two most disparate models.

Given the strong temperature-dependence of aggregation, we were able to use four climate projection models to generate predictions about how social behavior will respond to long-term changes in average temperature. By modeling monthly aggregation levels from 1950–2099, we found that the predicted proportion of the population aggregating each winter decreased over time both in peak magnitude and in the number of months per year with high levels of aggregation (Figure 4A). By 2099, annual aggregation is predicted to decline by a minimum of 20% to more than 50% of 1950 levels (four model mean = 33%), depending on the severity of the climate model (Figure 4B).

## Discussion

Our study provides strong empirical evidence that direct fitness benefits favor social aggregation and offers a clear mechanistic explanation for the formation of kin groups through juvenile philopatry [20]. Juvenile lizards are disproportionately favored to join winter groups because of their small size, but the fitness and thermal benefits of aggregation are evident in all age and sex classes. However, our results also suggest that aggregated males, females, and juveniles may have multiple ways of benefiting in this system. Our finding that aggregated lizards have higher fitness could be explained either by social interaction driving fitness benefits or by the preferential aggregation of otherwise high fitness individuals. The former interpretation better fits the observed data because the ability of aggregations to increase thermal buffering and reduce metabolic costs uniquely accounts for both the temperature dependence of group living and the absence of body condition differences in males. Both juveniles and females have special body condition needs not experienced by adult males; juveniles are challenged by their tiny size (average mass = 0.25 g) and high ratio of surface area to volume, and viviparous females by their high reproductive investment in litters up to half their body mass [26]. However, males may also use aggregations as a form of mate-guarding and be better able to retain female aggregation mates, accounting for the extreme reproductive skew towards social males. These two processes are not necessarily mutually exclusive, and they may well form a positive feedback loop promoting the maintenance of aggregative behavior even after the initial benefit to philopatric juveniles.

This research has implications for appreciating the potentially large ecological consequences of environmental variation that can occur when animal behavior is mediated by temperature. Potential and realized effects of climate change on species diversification [30], extinction [31], geographic range or niche shifts [32], and reproductive phenology [33] have been thoroughly discussed, but purely behavioral responses to climate change have received less attention (except for migration [34]). However, temperature-mediated behavior is present in many animal systems, of which huddling at cold temperatures is especially common [35], [36], [37], suggesting that our results are part of a more general phenomenon. In line with what we describe here in *Xantusia*, aggregation is considered beneficial in other winter huddling species because of the thermal advantages of buffering against extreme temperatures, although exact mechanisms vary slightly due to the differences in metabolic profiles between endotherms and ectotherms. Indeed, winter aggregation in some lizard species is also suggested to be mediated by thermal buffering [38], [39], although it should be noted that other species seem to have quite extensive social interactions without significant thermal pressures (especially in the Australian skink genus *Egernia*, reviewed in [9]).

The major question for species with temperature-mediated social aggregation is the ecological and behavioral response to broad scale changes in climate. Although our data suggest that there may be additional benefits to aggregation other than simple thermal buffering at cold temperatures (*e*.*g*., male reproductive success), the strong temperature dependence of social behavior (Figure 2) means that a mechanism is already in place that yields less aggregation at warmer temperatures. For annual aggregation to be maintained at current levels under future climate scenarios, this established relationship between temperature and group formation would have to break down. Moreover, several recent studies have implicated a strong environmental effect on macroevolutionary patterns of sociality and facultative social behavior [23], [40], [41], and changes in either behavior or ecology should be expected when the benefits of sociality are dependent on particular environmental conditions. Recognizing that simple changes in temperature may have profound and cascading effects on higher-order processes like social evolution is critical to appreciating the ramifications of future environmental change.

From a macroevolutionary perspective, investigating the transition between solitary and group living may inform the ramifications of extended conspecific interactions in the broader context of the evolution of sociality. The popular perception of social evolution is one of a unidirectional “evolutionary trajectory,” a progressive process in which species get locked into obligate sociality. Although this model is likely true for some systems (particularly eusocial insects), our data support the idea that social group formation may be a very dynamic process in species that maintain reproductive independence of individuals [16], including repeated bidirectional transitions between social and solitary states [42]. Especially in the early stages of sociality, transitions between social and solitary behavior may occur easily and rapidly by tracking changes in an ecological or environmental variable like temperature. Behavioral modifications are likely to be the first changes seen when environmental conditions vary through time, and this study adds to a growing body of work highlighting the importance of social plasticity and the role of environment in social evolution. Broadening such perspectives can guide both theoretical and empirical research by generating testable predictions about how, why, when, and in which species social behavior may arise and change over time and space.

## Methods

### Ethics Statement

All methods were approved by the Chancellor’s Animal Research Committee at the University of California, Santa Cruz (Sine00.02-1) and California Department of Fish and Game (SC-05560 to ARDR).

### Field Collection

We conducted a mark-recapture survey of 2,332 lizards from 441 social groups every summer and winter from August 2003 - January 2008 on a 36 hectare plot in the western Mojave Desert, approximately 16 km from Pearblossom, CA, USA (UTM coordinates easting 434561, northing 3816412, zone 11N). We hand-captured lizards by turning every fallen Joshua tree (*Yucca brevifolia*) log in this plot once per season.

Each winter, we classified any lizards found within a radius of approximately 30 cm of each other as aggregated. Although the vast majority of aggregated lizards were found in direct physical contact and unambiguously intertwined with the other group members, this guideline was occasionally necessary to account for the fleeing of lizards due to the unavoidable effects of sampling disturbance incurred by the rolling of logs (see Figure S1; [8]). For reference, this distance corresponds to approximately 3.5 times the total length of an adult *X. vigilis* and to the distance an adult can sprint in 0.5–1.5 seconds, even at cold body temperatures ([43]; Supporting Information). Although we think it is important to report that this guideline was used, we rarely needed to employ it and do not believe that it had a significant effect on our results or interpretation.

At each capture or birth, we measured the mass, snout-vent length (SVL), and tail condition (broken, regrown, intact) of each lizard. We sexed each lizard by shining a light through the base of the tail to visualize hemipenes in males [44]. We then toe-clipped each new individual for future identification and took a small piece of tail tissue (stored in 95% ethanol) for genetic analysis of paternity.

### Reproductive Success

To assess the reproductive success of social and solitary lizards, we analyzed only adults for which we knew aggregation status in the winter directly preceding summer reproduction (*N* = 34 females, 24 males, all from separate aggregations). Summer-recaptured females were kept in the laboratory until parturition to determine litter size and then returned with their offspring to their exact log of capture. We used general linear models (GLMs) to test for the potential confounding effect of body size (SVL) on litter size (*P* = 0.56) and to assess the effect of winter sociality on litter size.

To assign paternity, we genotyped 369 females, 249 males, and 624 juveniles at seven unlinked, highly polymorphic microsatellite loci [8]. We assigned paternity to candidate sires with pair or trio critical LOD scores above the 80% confidence level in Cervus v3.0.3 and included genotypes of known mothers when available. Although we were able to confidently assign paternity of 230 juveniles to 123 sires, only 24 of these males were also captured in the winter directly preceding offspring birth. Because all 24 of these sires were aggregated males, we used a binomial test with probabilities equal to the observed frequencies of the two aggregation states (69% of winter-captured males were aggregated) to assess the effect of winter sociality on male reproductive success. There was no significant effect of male body size (SVL) on reproductive success (*P* = 0.36).

### Survival

To estimate survival of social and solitary lizards, we used multi-state models in Mark v6.0 by coding encounter histories as A (solitary state), B (aggregated state), or 0 (not captured) over the 10 sampling occasions (*N* = 723 females, 443 males, 1166 juveniles). We constructed four main models with constant but state-specific survival (*Φ*_A_, *Φ*_B_), time-dependent state transition probabilities (*Ψ*_A_, *Ψ*_B_), and all four combinations of constant and time-dependent capture probabilities by aggregation state (pA, pB). To minimize over-parameterizing models, we independently analyzed adult males, adult females, and juveniles. Our sampling occasions were not equally spaced across the year, as winter observations were taken in December/January and summer observations in August/September during late stages of female pregnancy (see Methods for Reproductive Success). We accounted for these unequal time intervals by calculating the fraction of one year that had passed between the last day of the earlier sampling occasion and the first day of the later sampling occasions, and then scaling them by dividing each by the longest duration between sampling occasions. To account for aggregation (State B) only being present in the winter, we fixed eight of the possible 18 state transition probabilities (*Ψ*): *Ψ*_A→B_ was fixed to 0 and *Ψ*_B→A_ was fixed to 1 for all four winter to summer transitions.

We ran all models with the logit link function, 2ndPart variation estimation, and identity design matrix options. We then weight-averaged the real survival parameter estimates from our four models to obtain robust estimates of survival (Table S1) and compared solitary and aggregated survival estimates and error using CONTRAST [45]. We also performed likelihood ratio tests comparing the model with the lowest AIC score to the most reduced model (all parameters constant), and in all cases, the best fit model was significantly better than the reduced model (females: χ^2^ = 113.3, df = 11, *P*<0.0001; males: χ^2^ = 42.6, df = 3, *P*<0.0001; juveniles: χ^2^ = 101.5, df = 9, *P*<0.0001).

### Body Condition

To compare body condition of aggregated and solitary lizards, we log transformed (for linearity) mass and SVL of all winter-caught lizards at time of capture and regressed log mass against log SVL by age/sex class (*N* = 640 females, 389 males, 507 juveniles). We then used residuals from class-specific regressions as an index of body condition and used linear models to compare aggregated and solitary lizards within each class. All individuals with incomplete tails were excluded from analysis, and pregnancy does not occur in the winter.

### Environmental Temperature

We obtained historical temperature data for Pearblossom, CA, USA by downloading daily and monthly temperatures for the Pearblossom weather station (PWS; #046773) from the NOAA National Climatic Data Center (NCDC) database for all available years (1986–2009). We compiled summary statistics for annual number of nights below 0°C and annual extreme T_min_ for both the full 24 year period for which data were available and the 2003–2008 period during which we conducted our study (Table S2).

To assess the effect of field temperature on aggregation, we averaged maximum and minimum daily temperature data for each collection day from the PWS. We analyzed only days for which we caught more than 30 lizards to avoid small sample biases (N = 17 winter and 9 summer days). We then fit a self-starting three parameter logistic model to generate predicted aggregation values by temperature and used a Spearman rank test on the 17 winter collection days to assess the effect of temperature on winter aggregation.

### Laboratory Measurements of Heat Flux

To quantify the thermal stability of social groups, we measured heat flux of 26 naturally solitary lizards, 55 natural aggregations, and 27 experimentally isolated lizards originally found in aggregations. We placed lizards inside 59 ml cylindrical airtight plastic arenas filled across the bottom by a 2 cm diameter heat flux disk (Thermonetics, Inc.; Figure S5). We then placed these arenas inside an environmental chamber for at least two hours at 16°C before the temperature dropped to 1°C for 12 hours to simulate natural daytime and nighttime winter temperatures. Each heat flux disk was connected to an external data logger (Hydra®) that scanned all arenas once every six seconds and recorded heat loss curves for each arena each night. We also weighed lizards before and after each run to measure evaporative water loss (Text S1; Fig S3B). To compare cooling rates of aggregated and solitary lizards, we averaged measurements from each arena over one minute intervals, calculated the half-lives and rate of decay (by linearizing) for each of these classic first-order decay curves (Figure S5), and used GLMs to assess the effect of mass and aggregation. Although the cooling rate of this experiment is much faster than the real rate of environmental cooling, we could combine these lab-measured heat loss rates (lizard and environmental) with field-measured cooling rates (environmental; Text S1) to extrapolate natural rates of lizard heat loss and calculate the magnitude (mean and s.d.) of thermal buffering derived by juveniles (<0.3 g) joining aggregations (>4 g) in nature.

### Climate Models

We used LLNL-Reclamation-SCU downscaled climate projections data derived from the World Climate Research Programme’s (WCRP’s) Coupled Model Intercomparison Project phase 3 (CMIP3) multimodel dataset, stored and served at the LLNL Green Data Oasis. As per the setup of this online database’s access capabilities, we queried the closest geographic approximation of our field site, which was a 156 km^2^ rectangle encompassing our site in the middle of the rectangle’s eastern side. This downscaling method predicts average monthly temperatures for each of the four vertices of this rectangle, and we then averaged the values from the two northernmost vertices that were unambiguously in the desert instead of the foothills of the nearby San Gabriel Mountains (see Figure S1B) so as not to bias our temperature models towards elevations higher than our field site. We obtained projected mean monthly temperatures for two climate models (Centre National de Recherches Météorologiques (CNRM) CM3.1 and National Center for Atmospheric Research (NCAR) PCM1.1) under two different emissions scenarios (A2 and B1), yielding a total of four models.

### Modeling Social Behavior on Climate Projections

After acquiring average monthly temperature projections for the four climate models, we used the three parameter logistic model generated from observed aggregation by mean temperature (Figure 2) to predict monthly aggregation proportions for each model from 1950–2099. We then summed monthly aggregation proportions by year (area under yearly curves, Figure 4A) to create an Aggregation Index and fit logistic and linear models as appropriate to assess change in aggregation over time for each climate model (Figure 4B). Climate model CM3.1-B1 produced a linear relationship instead of a logistic curve. All four of these statistical models showed a significant negative relationship between cumulative annual aggregation and time as compared to model specific zero-slope lines (PCM1.1-B1: *ΔAIC* = 62.78; CM3.1-B1: *ΔAIC* = 83.71; PCM1.1-A2: *ΔAIC* = 115.15; CM3.1-A2: *ΔAIC* = 170.86).

### Statistical Analysis

Unless otherwise stated, we performed all statistical tests in R v2.13.1 and assessed significance at *P*<0.05. For parametric analyses, normality of residuals was assessed using Shapiro-Wilk tests, linearity by visual assessment of residual by predicted plots, autocorrelation with Durbin-Watson tests, and homogeneity of variance with Levene’s test for all relevant analyses. Nonparametric tests were chosen where described for data that violated assumptions of parametric analyses. Unless otherwise noted, only significant effects are reported.

## Supporting Information

Figure S1 **Night lizard aggregation and Joshua tree habitat.** (**A**) *In situ* night lizard (*Xantusia vigilis*) aggregation of three adults and two juveniles demonstrates winter huddling behavior. The lizard above is walking away from the aggregation after being disturbed by the rolling of the cover log. (**B**) Joshua tree (*Yucca brevifolia*) habitat at the field site shows both living trees and fallen logs, the site of winter aggregation.
(TIF)

Figure S2 **Fallen log microhabitat characteristics do not predict aggregation.** (**A**) None of the following habitat variables vary between sites with aggregations versus solitary lizards (left to right): Under log temperature (residual from regression with air temperature), buffer from air temperature, log decomposition (*p*-value from Chi square test of count data, excluding logs with no lizards), log size, nearest fallen log, or nearest living tree. (**B**) Evaporative water loss (EWL) rates are the same in aggregated and solitary lizards, and EWL rates do not scale with aggregation mass as expected if water loss needs were driving social behavior. Comparison graphs show means ±1 s.d.
(TIF)

Figure S3 **Daily temperature profiles underneath fallen logs.** Microhabitat temperatures measured by HOBO data loggers underneath five fallen logs are remarkably similar to environmental temperatures at the Pearblossom weather station, especially underneath preferred logs of medium decomposition (top row). Sheltering logs can weakly buffer lizards against extreme temperatures, but the maximum effect of this buffer is no more than a few degrees C and subzero temperatures are encountered as late as March.
(TIF)

Figure S4 **Lack of aggregation capture bias during daily temperature fluctuation.** (A) Typical temperature profile from sunrise to sunset (3 March 2002) from a HOBO data logger underneath fallen log of medium decomposition. (B) Group size by daily capture order (sunrise to sunset) show that lizards caught at the beginning of the day (the coldest temperatures) were not more likely to be aggregated. Aggregations were found throughout each collection day. Collection dates and average daily temperatures are at the top of each graph.
(TIF)

Figure S5 **Arenas for laboratory measurements of heat flux.** We measured heat flux in 26 naturally solitary lizards, 55 natural aggregations, and 27 experimentally isolated lizards originally found in aggregations by placing them inside plastic arenas filled across the bottom by a heat flux disk. We then placed these arenas inside an environmental chamber at 16°C before the temperature dropped to 1°C for 12 hours to simulate natural daytime and nighttime winter temperatures. We recorded changes in heat flux over time to generate heat loss curves for which we then calculated decay rate and half-life to compare the thermal stability of solitary and aggregated lizards.
(TIF)

Table S1 **Multi-strata survival model output from Mark.** All four main models for each class (female, male, juvenile), with constant survival (*Φ*) for each state (A  =  solitary, B  =  aggregated), time-dependent transition probabilities between states (*Ψ*_A→B_, *Ψ*_B→A_), but variable capture probability (p) parameters (t  =  time-dependent, .  =  constant). The weighted model averages are bolded, and the fully time-constant and time-dependent models are italicized for comparison. Estimates of survival are remarkably robust to changes in model structure; in all cases, the aggregated state is associated with higher survival than the solitary state.
(DOC)

Table S2 **Historical weather data for Pearblossom, CA, USA.** Annual average and extreme minimum temperatures and number of nights below freezing from 1986–2009 (subset 2003–2008 is the duration of this study). Source is NOAA National Climatic Data Center (NCDC) for the Pearblossom weather station (#046773).
(DOC)

Text S1 **Supporting text.**
(DOC)
