# Supplementary figures and images for: Direct Fitness Correlates and Thermal Consequences of Facultative Aggregation in a Desert Lizard
Source: PLoS One. 2012 Jul 23;7(7):e40866. doi: 10.1371/journal.pone.0040866 (PMC3402482; doi:10.1371/journal.pone.0040866)

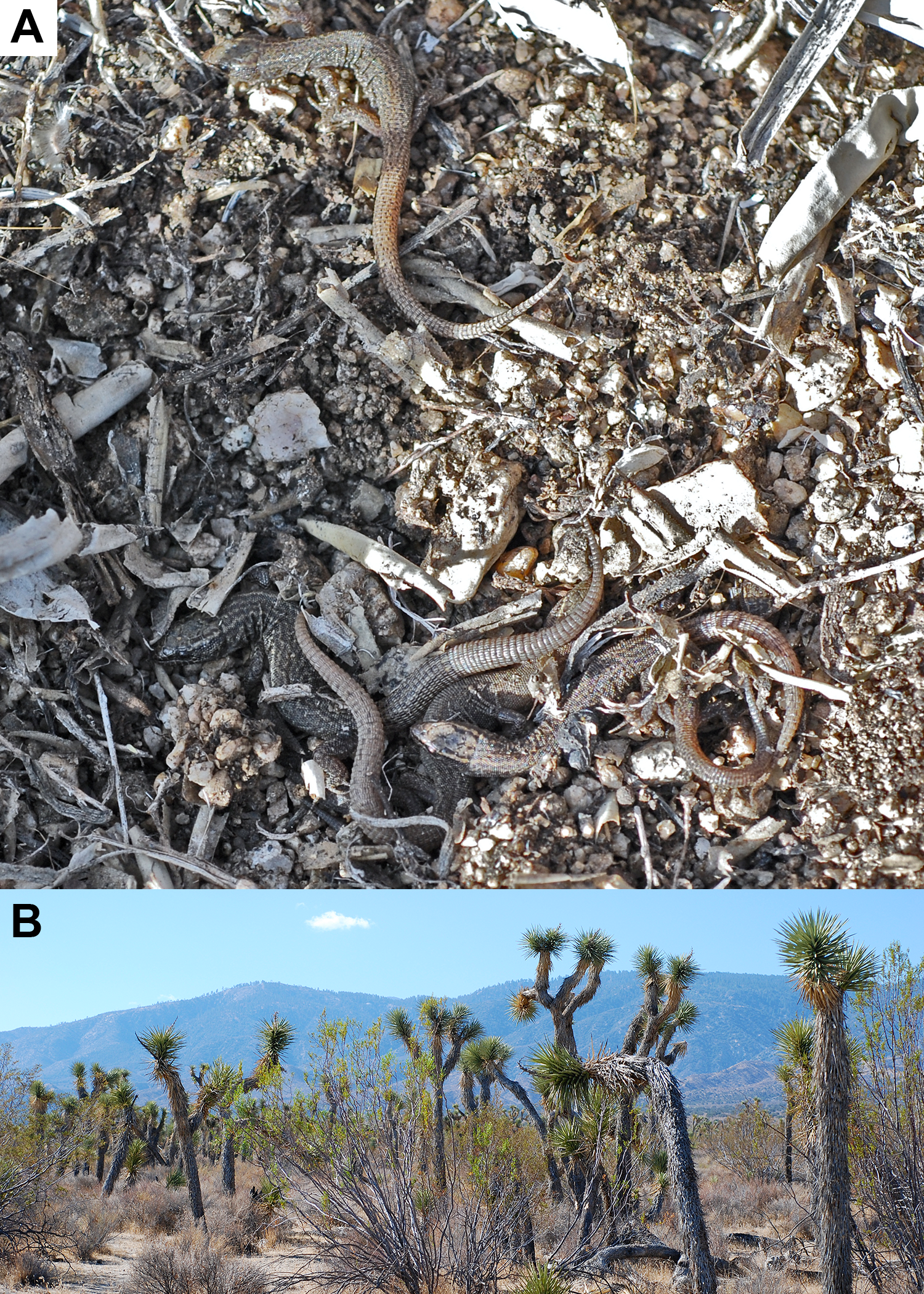

Supplement: Figure S1 — Night lizard aggregation and Joshua tree habitat. (A) In situ night lizard (Xantusia vigilis) aggregation of three adults and two juveniles demonstrates winter huddling behavior. The lizard above is walking away from the aggregation after being disturbed by the rolling of the cover log. (B) Joshua tree (Yucca brevifolia) habitat at the field site shows both living trees and fallen logs, the site of winter aggregation. (TIF) [file pone.0040866.s001.tif]

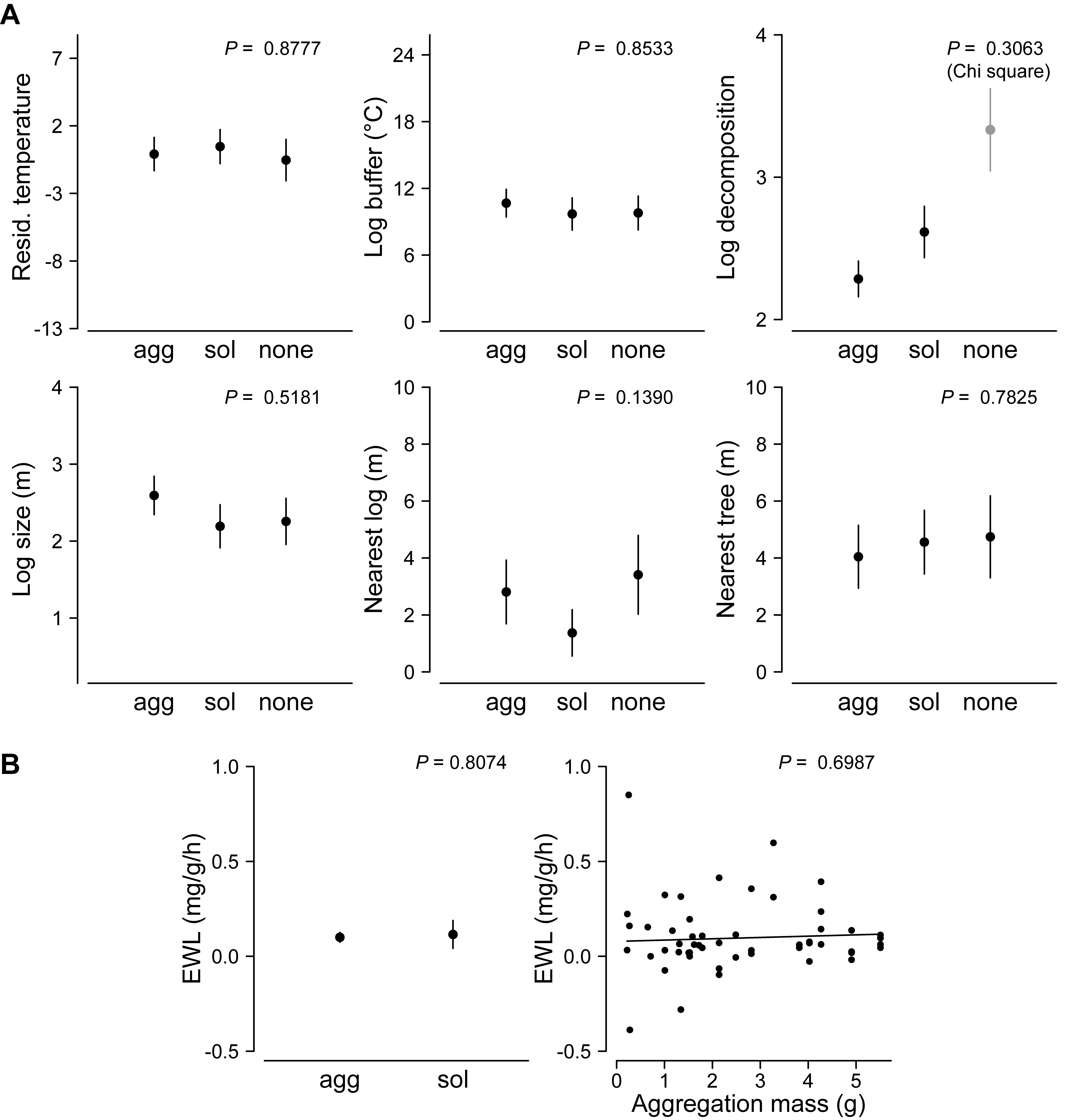

Supplement: Figure S2 — Fallen log microhabitat characteristics do not predict aggregation. (A) None of the following habitat variables vary between sites with aggregations versus solitary lizards (left to right): Under log temperature (residual from regression with air temperature), buffer from air temperature, log decomposition (p-value from Chi square test of count data, excluding logs with no lizards), log size, nearest fallen log, or nearest living tree. (B) Evaporative water loss (EWL) rates are the same in aggregated and solitary lizards, and EWL rates do not scale with aggregation mass as expected if water loss needs were driving social behavior. Comparison graphs show means ±1 s.d. (TIF) [file pone.0040866.s002.tif]

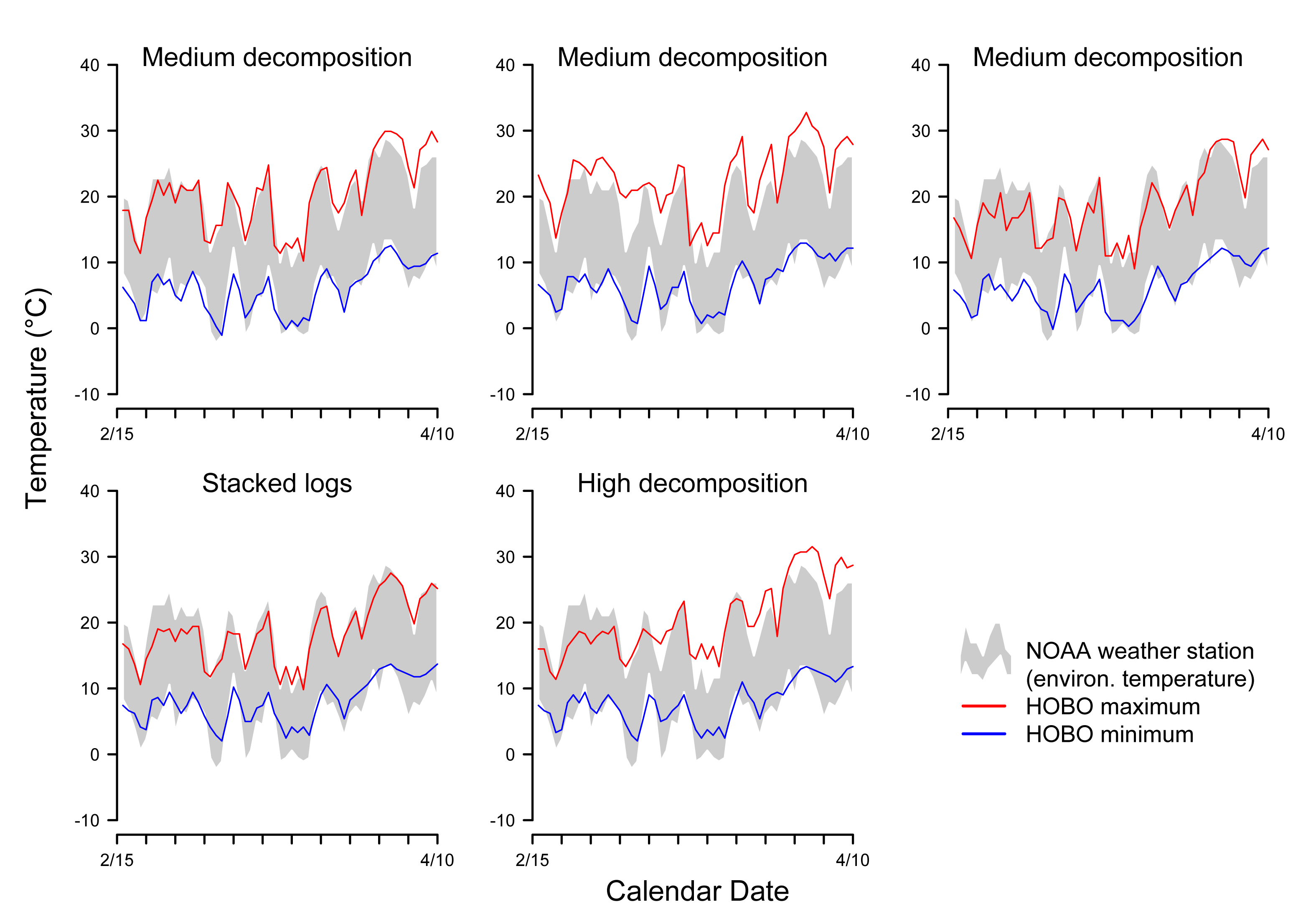

Supplement: Figure S3 — Daily temperature profiles underneath fallen logs. Microhabitat temperatures measured by HOBO data loggers underneath five fallen logs are remarkably similar to environmental temperatures at the Pearblossom weather station, especially underneath preferred logs of medium decomposition (top row). Sheltering logs can weakly buffer lizards against extreme temperatures, but the maximum effect of this buffer is no more than a few degrees C and subzero temperatures are encountered as late as March. (TIF) [file pone.0040866.s003.tif]

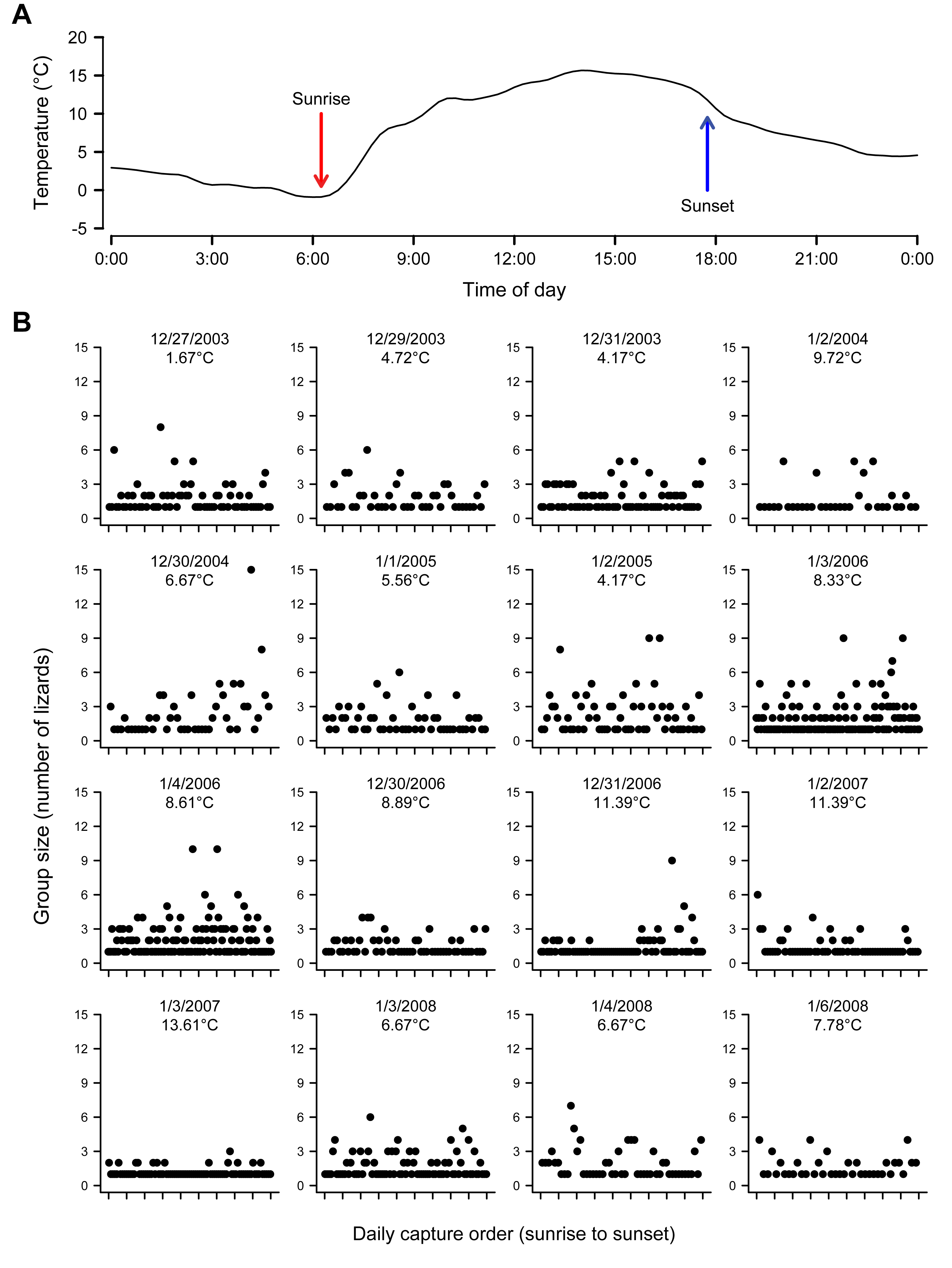

Supplement: Figure S4 — Lack of aggregation capture bias during daily temperature fluctuation. (A) Typical temperature profile from sunrise to sunset (3 March 2002) from a HOBO data logger underneath fallen log of medium decomposition. (B) Group size by daily capture order (sunrise to sunset) show that lizards caught at the beginning of the day (the coldest temperatures) were not more likely to be aggregated. Aggregations were found throughout each collection day. Collection dates and average daily temperatures are at the top of each graph. (TIF) [file pone.0040866.s004.tif]

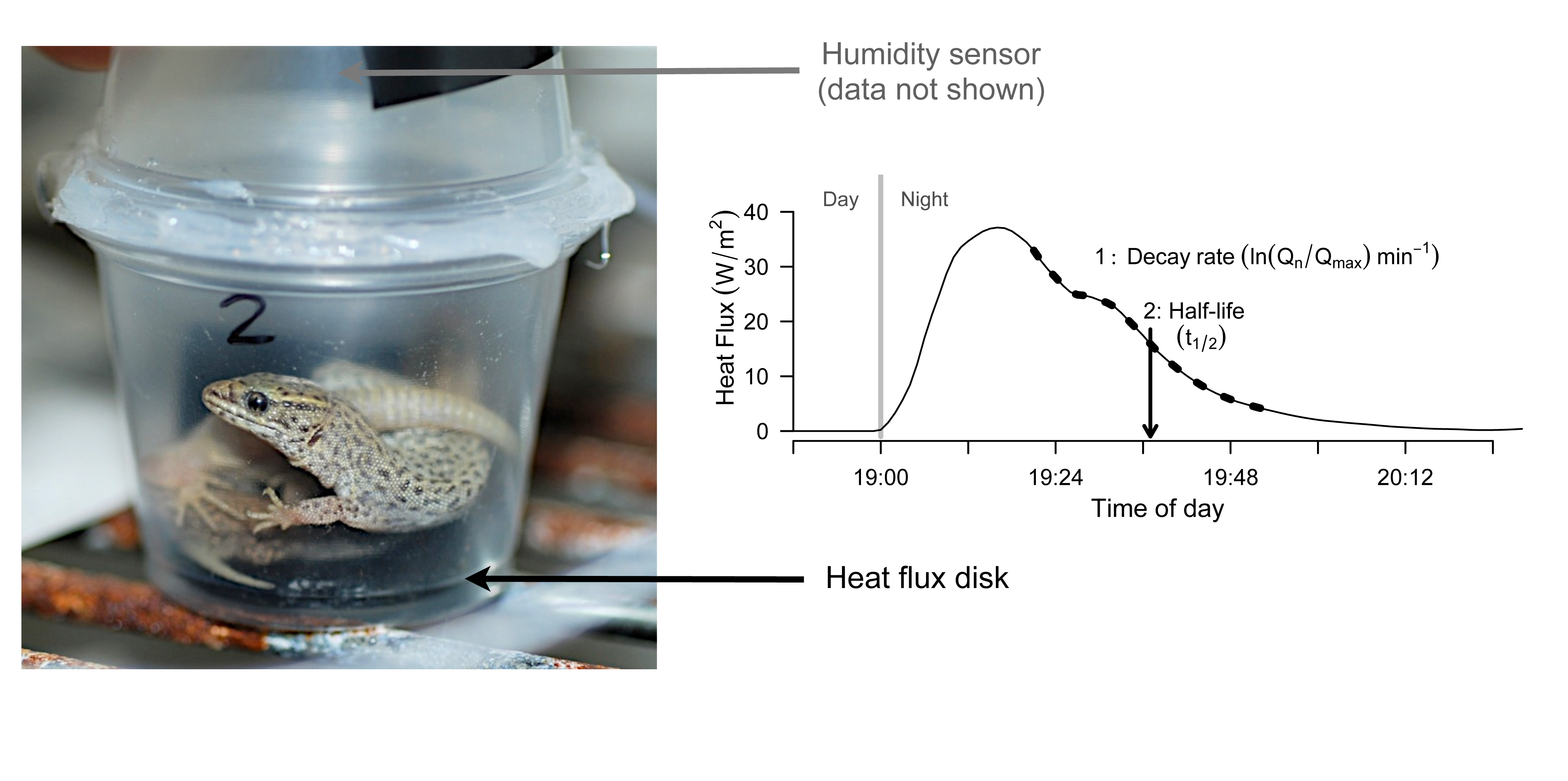

Supplement: Figure S5 — Arenas for laboratory measurements of heat flux. We measured heat flux in 26 naturally solitary lizards, 55 natural aggregations, and 27 experimentally isolated lizards originally found in aggregations by placing them inside plastic arenas filled across the bottom by a heat flux disk. We then placed these arenas inside an environmental chamber at 16°C before the temperature dropped to 1°C for 12 hours to simulate natural daytime and nighttime winter temperatures. We recorded changes in heat flux over time to generate heat loss curves for which we then calculated decay rate and half-life to compare the thermal stability of solitary and aggregated lizards. (TIF) [file pone.0040866.s005.tif]
