# Supplementary material for: Direct Fitness Correlates and Thermal Consequences of Facultative Aggregation in a Desert Lizard
Source: PLoS One. 2012 Jul 23;7(7):e40866. doi: 10.1371/journal.pone.0040866 (PMC3402482; doi:10.1371/journal.pone.0040866)
Supplement: Table S1 — Multi-strata survival model output from Mark. All four main models for each class (female, male, juvenile), with constant survival (Φ) for each state (A = solitary, B = aggregated), time-dependent transition probabilities between states (Ψ A→B, Ψ B→A), but variable capture probability (p) parameters (t = time-dependent, . = constant). The weighted model averages are bolded, and the fully time-constant and time-dependent models are italicized for comparison. Estimates of survival are remarkably robust to changes in model structure; in all cases, the aggregated state is associated with higher survival than the solitary state. (DOC) [file pone.0040866.s006.doc]

| *Class* | ***Model*** | ***# par*** | ***AIC*** | ***Weight*** | ***ΦB (aggregated)*** | ***SE*** | ***ΦA (solitary)*** | ***SE*** |
| --- | --- | --- | --- | --- | --- | --- | --- | --- |
| **Adult female** | pA(t)pB(.) | 16 | 1428.7881 | 0.5730 | 0.9316 | 0.1377 | 0.5936 | 0.1130 |
|  | pA(.)pB(t) | 10 | 1430.7610 | 0.2137 | 0.9406 | 0.1255 | 0.6136 | 0.0501 |
|  | pA(.)pB(.) | 8 | 1431.6550 | 0.1367 | 0.9378 | 0.1263 | 0.6009 | 0.0505 |
|  | pA(t)pB(t) | 18 | 1432.8095 | 0.0767 | 0.9186 | 0.1389 | 0.5771 | 0.1169 |
|  | **weighted average** | |  |  | **0.9334** | **0.1336** | **0.5976** | **0.0913** |
|  | *constant* | *5* | *1519.4863* | *0.0000* | *0.6753* | *0.0392* | *0.4174* | *0.0767* |
|  | *time-dependent* | *30* | *1439.7170* | *0.0024* |  |  |  |  |
| **Adult male** | pA(.)pB(t) | 8 | 709.6635 | 0.5170 | 0.8823 | 0.1672 | 0.6075 | 0.0684 |
|  | pA(.)pB(.) | 7 | 709.8363 | 0.4742 | 0.9464 | 0.1994 | 0.6440 | 0.0631 |
|  | pA(t)pB(.) | 14 | 718.0248 | 0.0079 | 0.9223 | 0.1952 | 0.6284 | 0.0653 |
|  | pA(t)pB(t) | 16 | 722.2640 | 0.0010 | 0.9158 | 0.1964 | 0.6142 | 0.0951 |
|  | **weighted average** | |  |  | **0.9130** | **0.1827** | **0.6249** | **0.0659** |
|  | *constant* | *5* | *746.0966* | *0.0000* | *0.8381* | *0.1603* | *0.5699* | *0.0896* |
|  | *time-dependent* | *31* | *730.7944* | *0.0000* |  |  |  |  |
| **Juvenile** | pA(t)pB(t) | 14 | 1248.2058 | 0.7403 | 0.8184 | 0.1051 | 0.5783 | 0.0705 |
|  | pA(t)pB(.) | 14 | 1250.3041 | 0.2593 | 0.8137 | 0.1446 | 0.6627 | 0.0544 |
|  | pA(.)pB(t) | 7 | 1264.3044 | 0.0002 | 0.8160 | 0.1442 | 0.6901 | 0.0518 |
|  | pA(.)pB(.) | 7 | 1264.3044 | 0.0002 | 0.8160 | 0.1441 | 0.6901 | 0.0518 |
|  | **weighted average** | |  |  | **0.8172** | **0.1153** | **0.6002** | **0.0663** |
|  | *constant* | *5* | *1331.4269* | *0.0000* | *0.8187* | *0.1363* | *0.6385* | *0.0584* |
|  | *time-dependent* | *31* | *1260.0486* | *0.0020* |  |  |  |  |
