# Supplementary material for: Direct Fitness Correlates and Thermal Consequences of Facultative Aggregation in a Desert Lizard
Source: PLoS One. 2012 Jul 23;7(7):e40866. doi: 10.1371/journal.pone.0040866 (PMC3402482; doi:10.1371/journal.pone.0040866)
Supplement: Table S2 — Historical weather data for Pearblossom, CA, USA. Annual average and extreme minimum temperatures and number of nights below freezing from 1986–2009 (subset 2003–2008 is the duration of this study). Source is NOAA National Climatic Data Center (NCDC) for the Pearblossom weather station (#046773). (DOC) [file pone.0040866.s007.doc]

| Year | # Nights below 0°C | Extreme min. temp. (°C) |
| --- | --- | --- |
| 1986 | 6 | -2.2 |
| 1987 | 36 | -7.8 |
| 1988 | 30 | -5.0 |
| 1989 | 31 | -8.3 |
| 1990 | 48 | -13.3 |
| 1991 | 6 | -7.2 |
| 1992 | 30 | -4.4 |
| 1993 | 28 | -5.6 |
| 1994 | 29 | -3.9 |
| 1995 | 19 | -2.2 |
| 1996 | 31 | -4.4 |
| 1997 | 36 | -2.8 |
| 1998 | 32 | -7.8 |
| 1999 | 30 | -3.3 |
| 2000 | 20 | -2.2 |
| 2001 | 28 | -3.9 |
| 2002 | 31 | -5.0 |
| 2003 | 16 | -5.0 |
| 2004 | 20 | -6.1 |
| 2005 | 12 | -1.7 |
| 2006 | 27 | -3.9 |
| 2007 | 39 | -9.4 |
| 2008 | 32 | -6.1 |
| 2009 | 39 | -5.0 |
| 1986 - 2009 Average | 27.3 | -5.4°C |
| 1986 - 2009 Extreme | 48 | -13.3°C |
| 2003 - 2008 Average | 24.3 | -5.4°C |
| 2003 - 2008 Extreme | 39 | -9.4°C |
