# Supplementary material for: Direct Fitness Correlates and Thermal Consequences of Facultative Aggregation in a Desert Lizard
Source: PLoS One. 2012 Jul 23;7(7):e40866. doi: 10.1371/journal.pone.0040866 (PMC3402482; doi:10.1371/journal.pone.0040866)
Supplement: Text S1 — Supporting text. (DOC) [file pone.0040866.s008.doc]

Supporting Text for

**Direct Fitness Correlates and Thermal Consequences of Facultative Aggregation in a Desert Lizard**

Alison R. Davis Rabosky, Ammon Corl, Heather E.M. Liwanag, Yann Surget-Groba, Barry Sinervo

correspondence to: [ardr@umich.edu](mailto:ardr@umich.edu?subject=)

**Supporting Text**

*Measurements of habitat characteristics*

To exclude the possibility that lizards are simply aggregating in the “best” microhabitat sites due to limited or patchy resource distribution (food, water, and adequate shelter, reviewed in [1]), we assessed a number of environmental variables and compared them between sites with aggregated lizards, sites with solitary lizards, and sites with no lizards.

Before assessing how environmental variables are likely to affect aggregation in night lizards, it is helpful to consider the consequences and implications of 1) sociality restricted to winter, and 2) the ectothermic physiology of lizards. The limited distribution of food and water is an unlikely explanation for winter sociality in night lizards. Xantusiid lizards have extremely low energy requirements, as a 10 g Xantusiid would only have to eat 1/3 as much food as a 10 g *Sceloporus* lizard and 2% of the food required by a 10 g bird [2]. For reference, the consumption of one 0.02 g insect larva would fulfill the energy requirements of an adult *X. vigilis* (1.5 g) for 4 days [2]. Furthermore, food and water requirements drop to near zero in winter because metabolic and water influx rates in ectotherms slow down in colder temperatures (see [2] for measured values). This reduction is further offset by the Mediterranean climate of California, in which more than 80% of annual rainfall in the Western Mojave Desert occurs between November and March (see Figure 4 in [3]). If food and water were limiting factors, *X. vigilis* should aggregate in the summer when their energetic needs are the highest and when water is a limiting resource [4]. Additionally, stomach contents analyses suggest that Xantusiids have unusually diverse diets, consuming a wide variety of both plant and animal products [5], implying that they are not dependent on just one food resource whose distribution could drastically influence the lizards’ spatial pattern. Personal observations in the field support this as well, as high prey density termite nests are sporadically distributed with no effect on relative lizard density. However, strong reliance on cover objects and the naturally patchy distribution of fallen Joshua tree logs due do the spatial clumping of living trees suggest that characteristics of adequate shelter could be a limiting factor. It must be noted, however, that summer distributions are 1-2 lizards per log with no evidence of increased mortality despite low critical thermal limit (death at 38°C [6]), and that several other lizard species aggregate even when shelters are in excess (reviewed in [1]).

To assess differences in microhabitat quality among sites with no lizards, solitary lizards, and aggregations, we sampled every cover object in a predefined area at a neighboring field site to the main study area (N = 37 logs). For each fallen log, we measured log length, distance to nearest neighboring log, distance to nearest upright Joshua tree, and air temperature. We also characterized the log’s state of decomposition by ranking it on a scale of 1-4 from low to high (1: 0-10% of leaves gone; wood is rigid and hard to pull apart; 2: 20-50% of leaves gone; outer wood is light grey; 3: 60-80% of leaves gone; outer wood is smooth and weathered; 4: 100% of leaves gone; wood is spongy and disintegrates upon contact). After overturning the log, we measured the temperature underneath the log at the lizard’s known position if one was present, and temperature at the middle and both ends of the log if no lizards were found (averaged for analysis). Under-log temperatures were residualized against ambient air temperature to allow comparison of data taken at different times of day, but there was no effect of ambient temperature on overall log buffering capacity (buffer = T_ambient_ - T_log_; *P* = 0.347). To assess differences among logs with no lizards, solitary lizards, and aggregations, we used standard GLMs for all analyses except for log decomposition (Chi-square test on count data) and the two distance measurements (censored regression with a cutoff point at 10m).

We found that night lizards do seem to be constrained to logs with medium levels of decomposition retaining reasonable structural integrity, as very decomposed logs are more likely to have no lizards (Figure S2A). Within this given range of suitable habitat, however, we have found no microhabitat characteristics that differ between sites containing no lizards, solitary lizards, or aggregations (Figure S2A). Only one habitat variable seems to make one microsite more advantageous than another: the concomitant presence of other lizards.

Both temperature and evaporative water loss (EWL) have been suggested to drive sociality and investigated in other lizard species [4,7], although these studies have never been paired with fitness measurements in natural populations. Despite the fact that winter is not the season of peak water stress (see above), we considered both during the laboratory measurements of heat flux (see Methods). The significant thermal benefits to aggregation are reported in the main manuscript (Figure 3), but we found no difference between rates of EWL (mg water loss per g body mass per hour) in aggregated and solitary lizards (Figure S2B). Additionally, EWL rates were not dependent on aggregation mass, which would have been expected if water loss needs were driving social behavior (Figure S2B). This result, and the low overall rates of EWL, are unsurprising given the very low respiration rate of lizards at temperatures near 0°C (and thus little opportunity for water loss to occur; [8]).

*Predation*

Predation was not directly addressed in this study because all known predators of this lizard are ectotherms with temperature-dependent activity (large invertebrates [3], other lizards [9], and snakes [10]). The limited activity patterns of such predators at cold temperatures suggest that aggregation benefits due to reduced predation risk could not explain winter-restricted sociality in night lizards, and especially not increased aggregation at the lowest temperatures. The high dependence of *X. vigilis* on well-fortified fallen *Yucca* logs for cover, low dispersal levels, and lack of any visible log disturbance during this study combine to suggest little opportunity for mortality via endothermic avian or mammalian predators that could be active at cold temperatures.

*Relationship between external and microhabitat temperature*

To more thoroughly assess the difference between environmental temperature from the Pearblossom Weather Station (see main text Methods) and microclimate at sites of lizard aggregation (log buffering capacity), we obtained daily temperature profiles from underneath fallen Joshua tree logs at our field site. From 15 February to 14 April 2002, we placed HOBO® data loggers underneath five fallen logs of different structural characteristics: three logs of medium decomposition (one of them a known aggregation site), one log of high decomposition, and one set of logs that were naturally stacked upon each other (Figure S3). Data loggers recorded temperature every 15 minutes over the 55 day sampling period, and there were no significant differences in temperature readout among loggers when exposed to the same temperature. We found that microhabitat temperatures underneath all log types are remarkably similar to environmental temperatures at the Pearblossom weather station (Figure S3). Sheltering logs, especially those of the preferred medium decomposition level, can weakly buffer lizards against extreme temperatures, but the maximum effect of this buffer is no more than a few degrees C and subzero temperatures are encountered underneath logs as late as March. These results further support the conclusion that all available sites within the microhabitat experience the same cold temperatures and that there is nothing inherently special about the sites chosen for aggregation, except the presence of other lizards.

Additionally, we conducted a small-scale study measuring lizard body temperature during winter collection in January 2007 using a Raytek Raynger PM infrared temperature sensor (data not shown). After nights that fell below freezing, we found that lizards had surface temperatures (a reasonable measure of body temperature due to the lizards’ small size) of -0.5 – 0°C in the early morning and that lizards at these low temperatures were surprisingly ambulatory.

*Tests for collection bias*

To test for an effect of daily fluctuations in temperature on the frequency of finding of aggregations, we regressed group size by capture order for each winter collection day (Figure S4). Although we would predict a negative relationship if aggregation behavior was based on small-scale variation in external temperature (with groups caught disproportionately in the cold mornings rather than the warm afternoons), none of these regressions had a slope different from zero (*P* > 0.05).

*The importance of thermal buffering through aggregation: Freeze tolerance in lizards*

We found clear evidence that sociality is correlated not only with increased reproductive success and survival, but that the formation of these groups is tightly linked to cold temperature and that grouped lizards have slower rates of heat loss. Slowing this rate by aggregating with conspecifics likely has important consequences for tolerating cold winter temperatures, and subsequently for both survival and reproductive success. This effect is likely the strongest when temperatures drop below 0°C.

Because lizards lack ice-structuring proteins like those found in Antarctic fish [11], most species (including *X. vigilis* [12]) cannot tolerate even limited ice crystallization inside the body [8]. Ice formation often results in severe and permanent neuromuscular problems before death within a few days [12-14]. Lizards that do survive a freeze event often require days or weeks to recover, and even the highly freeze-tolerant Common Lizard (*Lacerta vivipara*) cannot breathe for four hours after thawing [13]. Recovering a simple righting reflex (flipping to the stomach after being placed on the back) takes almost two days [13]. This recovery after a freeze event involves metabolically expensive tissue repair and severe oxidative stress [15].

The most common mechanism lizards employ to avoid freezing when subjected to subzero temperatures is to supercool the body, in which the tissue temperature drops below its freezing point without the formation of ice crystals. Supercooling ability has been identified in at least 42 lizard species distributed across phylogeny [8,12,14] and most likely occurs via increased blood concentrations of low molecular weight compounds like glucose or lactate [15-17]. Although individual *X. vigilis* have an average freezing point of -0.58°C and lizards can and do freeze at this temperature, some individuals are able to supercool their bodies to an average of -5.86°C before ice crystallization begins [12] and survive moderate subzero temperatures. However, supercooling can be a risky strategy, as any ice nucleation event within a supercooled liquid results in rapid and extensive crystallization throughout the body (accompanied by a temperature spike back up to freezing point [14]). Perhaps unsurprisingly, there is a striking similarity between the measured supercooling temperature limit of *X. vigilis* (-5.86°C) and the average extreme minimum temperature of the Pearblossom field site (-5.4°C, Table S2).

Slowing the rate of heat loss by aggregating with conspecifics can increase lizard fitness in two main ways. First, cooling too fast can reduce or even eliminate a lizard’s ability to supercool [14,18], presumably due to stimulation of ice nucleation through development of thermal gradients within the body [17] or to a time lag in the mobilization of blood glucose [16]. Second, tight aggregation between lizards serves to increase a lizard’s “body mass” and decrease the rate of freezing, as objects of greater mass freeze more slowly than smaller ones (parallel to the mass effect on heat loss shown in Figure 3). Slowly-cooling aggregated lizards will simply take longer to freeze than solitary lizards. Even if aggregated lizards were to freeze at the same rate as solitary lizards once they hit their freezing point, aggregations will still take longer to reach that freezing point itself and spend less time at dangerous temperatures (Figure 3E). This strategy is an excellent way to thermally benefit from greater body mass without a concomitant increase in each lizard’s blood volume, because larger blood volumes are positively correlated with probability of ice nucleation [19]. Reducing the amount of time spent at sub-zero temperatures will by definition reduce the chances of death or injury by freezing, directly increasing survival and allowing more energy to be spent on metabolic processes other than tissue repair. This additional energy would be especially important for juveniles because of their very small size and lack of stored energy, but also for adult females for viviparous reproduction. Lizards with fewer metabolic demands may be able to better recover after a cold freeze event, perhaps explaining why we found a more marked effect of sociality on body condition and survival in juveniles and females than in males.

**Supporting References**

1. Elfstrom BEO, Zucker N (1999) Winter aggregation and its relationship to social status in the tree lizard, *Urosaurus ornatus*. Journal of Herpetology 33: 240-248.

2. Mautz WJ (2000) Xantusiid lizards have low energy, water, and food requirements. Physiological and Biochemical Zoology 73: 480-487.

3. Zweifel RG, Lowe CH (1966) The ecology of a population of *Xantusia vigilis*, the Desert Night Lizard. American Museum Novitates 2247: 1-57.

4. Lancaster JR, Wilson P, Espinoza RE (2006) Physiological benefits as precursors of sociality: why banded geckos band. Animal Behaviour 72: 199-207.

5. Brattstrom BH (1952) The food of the night lizards, genus *Xantusia*. Copeia: 168-172.

6. Cowles RB, Bogert CM (1944) A preliminary study of the thermal requirements of desert reptiles. Bulletin of the American Museum of Natural History 83: 265-296.

7. Shah B, Shine R, Hudson S, Kearney M (2003) Sociality in lizards: Why do thick-tailed geckos (*Nephrurus milii*) aggregate? Behaviour 140: 1039-1052.

8. Voituron Y, Verdier B, Grenot C (2002) The respiratory metabolism of a lizard (*Lacerta vivipara*) in supercooled and frozen states. American Journal of Physiology-Regulatory Integrative and Comparative Physiology 283: R181-R186.

9. Perkins M, Adolph SC, Granite S, Hein W (1997) *Xantusia vigilis* (Desert Night Lizard) and *Sceloporus magister* (Desert Spiny Lizard). Predation and Diet. Herpetological Review 28: 89.

10. Rodríguez-Robles JA, Mulcahy DG, Greene HW (1999) Feeding ecology of the Desert Nightsnake, *Hypsiglena torquata* (Colubridae). Copeia 1999: 93-100.

11. Knight CA, Devries AL, Oolman LD (1984) Fish antifreeze protein and the freezing and recrystallization of ice. Nature 308: 295-296.

12. Lowe CH, Lardner PJ, Halpern EA (1971) Supercooling in reptiles and other vertebrates. Comparative Biochemistry and Physiology 39: 125-135.

13. Voituron Y, Storey JM, Grenot C, Storey KB (2002) Freezing survival, body ice content and blood composition of the freeze-tolerant European common lizard, *Lacerta vivipara*. Journal of Comparative Physiology B-Biochemical Systemic and Environmental Physiology 172: 71-76.

14. Spellerberg IF (1972) Temperature tolerances of southeast Australian reptiles examined in relation to reptile thermoregulatory behavior and distribution. Oecologia 9: 23-46.

15. Rey B, Sibille B, Romestaing C, Belouze M, Letexier D, et al. (2008) Reptilian uncoupling protein: functionality and expression in sub-zero temperatures. Journal of Experimental Biology 211: 1456-1462.

16. Storey KB (2006) Reptile freeze tolerance: Metabolism and gene expression. Cryobiology 52: 1-16.

17. Costanzo JP, Lee RE (1995) Supercooling and ice nucleation in vertebrate ectotherms. In: Lee RE, Warren GJ, Gusta LV, editors. Biological Ice Nucleation and its Applications. St. Paul, MN: APS Press. pp. 221-237.

18. Lotshaw DP (1977) Temperature adaptation and effects of thermal acclimation in *Rana sylvatica* and *Rana catesbeiana*. Comparative Biochemistry and Physiology 56: 287-294.

19. Vali G (1995) Principles of ice nucleation. In: Lee RE, Warren GJ, Gusta LV, editors. Biological Ice Nucleation and Its Applications. St. Paul, MN: APS Press. pp. 1-28.
